# Violence tendency and internet addiction in adolescents

**DOI:** 10.1590/1806-9282.20240394

**Published:** 2024-09-13

**Authors:** Kübra Gökalp, Hatice Durmaz, Nurgul Karakurt, Eda Ay, Suha Gökalp

**Affiliations:** 1 Atatürk University, Faculty of Nursing, Department of Psychiatric Nursing – Erzurum, Turkey; 2 Erzurum Technical University, Faculty of Health Science, Department of Nursing – Erzurum, Turkey; 3 Atatürk University, Pasinler Vocational School, Department of Computer Technologies – Erzurum, Turkey.

**Keywords:** Psychology, Child, Adolescent psychiatry, Internet

## Abstract

**OBJECTIVE:**

The aim of this study was to determine the relationship between internet addiction and violence tendency in adolescent students and the factors affecting violence tendency.

**METHODS:**

The research was conducted on 2,648 middle school students in Turkey. A socio-demographic form, the Young Internet Addiction Scale, and the Violence Tendency Scale were used to collect data. The data were analyzed using descriptive statistics and multiple regression analysis.

**RESULTS:**

It was determined that there was a significant positive relationship between the Young Internet Addiction Scale and the Violence Tendency Scale. Age has a positive effect on violence tendency levels.

**CONCLUSION:**

These findings suggest that the variables of age and internet addiction contribute to the occurrence of violence tendency. Psychiatric nurses should plan trainings and evaluate its effectiveness to raise awareness.

## INTRODUCTION

There have been striking technological developments in the world since the second half of the twentieth century. One of these, the internet, has become a part of life^1^. While it has many advantages, such as providing fast communication, saving time, offering access to sufficient and satisfying information, and shortening distances between people, it also brings many sociological and psychological problems when not used consciously^1,2^. One of these problems is internet addiction. Internet addiction leads to negative consequences such as mental illness and an aggressive attitude^3^. Many children and adolescents can use the internet for harmful content, including violence and pornography^4^.

Violence is a widespread public health problem in the world, like internet addiction. Violence negatively affects children's learning processes, mental health and development, quality of life, and levels of success^5-7^. Many types of negative events lead to a lower threshold for violence, including the easier expression of emotions in a computer environment than in face-to-face communication, the content of sites on the internet and experiencing failure in games, ignoring chat site negative events, offensive emails and jokes, and sexual requests, which lead to frequent exposure to violent images^4^. Frequent exposure to violent images causes the spread of violence and increases insensitivity to violence in society^8^.

In the literature, there are studies examining the internet and violence separately and together. However, these studies have mostly focused on high school and university students with a high tendency toward violence and internet addiction^7,9^. In the literature, studies found that internet addiction was positively associated with aggressive behaviors and violence tendency in grades 9–12 students^5,10^.

This study was undertaken to implement preventive measures before students’ internet addiction and violence trends reach their highest point, which is important. In addition, there is no study with a large sample group of fifth- and eighth-grade adolescents. For these reasons, this descriptive study was designed to determine the relationship between internet addiction and violence tendency in a large sample of students studying in middle school, as well as the factors affecting it.

Research questions:

1. What are the levels of violence tendency and internet addiction?
2. Do the levels of violence tendency and internet addiction have sociodemographic variables in adolescents?
3. Is there a relationship between violence tendency and internet addiction in adolescents?
4. Does an adolescent's age and age to start using the internet affect the level of violence tendency?
5. Does internet addiction affect the level of violence tendency in adolescents?

## METHODS

### Design and sample

This descriptive research was conducted between September 2018 and February 2020 in a province in the east of Turkey. When reporting the paper, we used the The Strengthening the Reporting of Observational Studies in Epidemiology (STROBE) guidelines^11^.

There are three districts in the center of the city where the research was conducted. There are 30 middle schools and 15,312 students in these three districts. To ensure a sufficiently large sample size, it was decided that at least 880 students from each of the three districts should be included (p=0.5, q=0.5). However, since a single school population did not meet this sample size, the two schools with the largest student populations were included in the study. The study was carried out on 2,648 students, out of the 3,258 students.

The criteria for inclusion in the research were as follows:

- The family gave their consent.
- The child was 11 years old or older.
- The child had no auditory, visual, or mental disabilities.
- The child was willing to participate in the study.

### Data collection instruments

Immediately following the end of a lesson in the classroom of the relevant school, the students who were included in the research were given the questionnaire by the researchers, who explained the process to them.

Socio-Demographic Form: This form consisted of two questions covering students’ personal information and three questions covering students’ internet usage.

Young Internet Addiction Scale (YIAS): The scale created by Young has 20 questions^12^. During the research of Bayraktar, the scale that had been translated from English to Turkish was adapted in such a way that adolescents could understand the items without disturbing the integrity of the items^13^. The reliability of the translation test in that study, in terms of standardized Cronbach's alpha, was 0.91. In this study, Cronbach's alpha was 0.92.

Violence Tendency Scale (VTS): This scale was developed by Göka, Bayat and Türkçapar in 1995 on behalf of the Ministry of National Education and in the study of the Prime Ministry Family Research Institution on ‘Violence in Family and Social Sphere’ (1998) in order to measure the violence tendencies^14^. The VTS consists of 20 items, and since all items are one-sided, there are no reverse-scored items. Therefore, the high scores obtained from the scale indicate that the tendency to violence is also high. The Cronbach's alpha reliability coefficient measured at different times on the scale was 0.78 and 0.87. In this study, Cronbach's alpha was 0.86.

### Statistical analysis

The data were analyzed with the IBM SPSS V-26 software. The normal distribution of variables was examined. Descriptive statistics were used for the demographic characteristics of the individuals. To evaluate the data, percentages, means, independent samples t-test, Pearson correlation, regression analyses, and Cronbach's alpha analyses were used.

## ETHICS

Permission was obtained from the university ethics committee and the institutions to collect the data for the study. The necessary explanations were made regarding the students participating in the study. During the data collection process, the researchers informed the participants about the study. After participants and their families were given written consent, the data were collected.

## RESULTS

The participants in the study had a mean age of 12.45±1.16 years, and 51.1% were male. It was also determined that the mean age to start using the internet was 8.52±2.00 years. Additionally, 44.9% of the participants owned smartphones, and 60.8% used the internet for learning.

The mean YIAS scores of the students were 25.30±19.57 (min=0, max=100). The mean VTS scores of the students were 39.64±10.83 (min=20, max=80) (Table 1).

**Table 1:** Distribution of students by demographic characteristics.

Demographic characteristics (n=2,648)		VT	IA
Gender			
	Female	38.42±10.68	21.77±18.73
	Male	40.81±10.85	28.68±19.77
	t value	−5.760**	−9.222**
Smartphone status			
	Yes	41.49±11.12	30.11±20.49
	No	38.11±10.37	21.39±17.85
	t value	7.974**	11.698**
Internet use for			
	Learning	37.04±9.85	17.94±14.60
	Game, video, and social networking	43.69±11.06	36.75±20.79
	t value	−16.143**	−27.324**
Mean scores		39.64±10.83	25.30±19.57

** p≤0.001.

When violence tendency and internet addiction were examined according to the demographic status features (Table 1), it was found that the average VTS and YIAS scores of the boys were higher than those of the girls (p≤0.001). The results showed that the VTS and YIAS scores of students who have smartphones were higher than those who do not. The difference between the average VTS and YIAS scores and having a smartphone was statistically significant (p≤0.001). It was found that the VTS and YIAS scores of participants who use the internet for games, videos, and social networking were higher than those who use the internet for learning (p≤0.001) (Table 1).

The correlations between the violence tendency and internet addiction (r=0.574; p≤0.001) were significantly positive (Table 2). In addition, correlations between violence tendency and age (r=0.182; p≤0.001) and age to start using the internet (r=-0.099; p≤0.001) were found to be significant (Table 2).

**Table 2:** Correlations values of study variable.

	1	2	3	4
WT (1)	1			
YIAS (2)	0.574**	1		
Age (3)	0.182**	0.229**	1	
Age to start using the internet (4)	−0.099**	−0.185**	0.285**	1

** p≤0.001.

Hierarchical regression analysis was performed to determine the effect of demographic variables age, age to start using the internet, and internet addiction level on the violence tendency of the students (Models 1 and 2).

As a result of the analysis:

### Model 1

In this study, it was determined that independent demographic variables (age and age to start using the internet) predicted 5.8% of the variance in violence tendency (Adj. R^2^=0.058; F=81.252; p≤0.001) (Table 3). Age (β=0.229; p≤0.001) and age to start using the internet (β=-0.164; p≤0.001) were found to be significantly related to violence tendency. Accordingly, as the age of the students increased, the mean of the VTS score increased. Another finding was that as the age of the students to start using the internet decreased, the mean of the VTS score increased.

**Table 3:** Hierarchical regression model for Violence Tendency Scale.

Dependent variable Violence Tendency Scale						
Independent variables		B	β	t	p	
Model 1:						Adjusted R^2^=0.058 R^2^ change=0.057 F=81.252
	(Constant)	20.596		9.353	0.001**	
	Age	2.123	0.229	11.628	0.001**	
	Age to start using the internet	−0.867	−0.164	−8.323	0.001**	
Model 2: Predictor variables						Adjusted R^2^=0.333 R^2^ change=0.332 F=439.174
	(Constant)	25.694		13.812	0.001**	
	Age	0.534	0.058	3.316	0.001**	
	Age to start using the internet	−0.062	−0.012	−0.683	0.495	
	YIAS	0.310	0.559	32.988	0.001**	

B: coefficient B; β: standardized beta coefficient; R^2^: R-square (the coefficient of determination);

** p≤0.001.

### Model 2

The results showed that independent variables (age, age to start using the internet, and YIAS) predicted 33.3% of the variance in violence tendency (Adj.R^2^=0.333; F=439.174; p≤0.001) (Table 3). Age to start using the internet (β=-0.012; p=0.495) was found to be insignificantly related to violence tendency. Another finding of the research was that VTS were positively affected by age (β=0.058; p≤0.001) and YIAS (β=0.559; p≤0.001). As the age and YIAS score of the students increased, the mean of the VTS score increased.

The regression equation that explained the correlation among the variables after Model 2 is shown below:

VTS=25.694+(0.534)Age−(0.310) Age to Start Using Internet+(0.310)YIAS

## DISCUSSION

This study draws attention to the relationship between violence tendency, internet addiction, and demographic variables. The violence affects students’ school success^6^, health problems^7^, quality of life, and the risk of diseases^15^. The violence tendency scores of the participants were moderate. Similar results were seen in studies related to this subject^5^. There were also studies showing that the violence tendencies of adolescents were higher than in this study^16,17^. Considering that both studies were conducted with high school students and the violence tendency among adolescents increases with age, it was an expected finding that the violence tendency mean scores were moderate in this research.

Today, violence and internet use are increasing, and the age at which children begin to use the internet has decreased to preschool age^18^. Internet addiction, like violence, is a public health problem as it decreases students’ school success and leads to health problems^19^. In this study, the internet addiction scores of the students were low. Feng et al. on middle-school students and Karaca et al. on high-school students determined a similar result^9,20^.

The results show that male adolescents had a higher violence tendency than female adolescents, which is in line with the findings of another study^21^. These findings may be associated with societies dominated by men that encourage stereotypical male behavior, such as displaying physical superiority and using brute force.

The research found that male students have a higher internet addiction than female students. Similar results have been seen in studies related to this subject^22^.

Students who have smartphones appear to experience a higher violence tendency and internet addiction than students who do not. Similar results have been found in other studies^23^. It was thought that the presence of a smartphone may lead to an increase in the level of addiction, as they offer more convenient, easier, cheaper, and on-demand access to the internet.

Another finding of the research is that the violence tendency and internet addiction of adolescents who use the internet for games, videos, and social networking were higher than those who use the internet for learning. It was thought that games, videos, and social networking may cause addiction by affecting hormones and a tendency toward violence due to their content.

In this study, age and age to start using the internet significantly affected violence tendency by 5.8% in Model 1. The finding of the research was that as adolescents’ ages increase, their violence tendency also increases. In a study with high school students, Altintas and Ayaz-Alkaya found that age affected violence tendency^17^. The results also show that as the age at which adolescents start to use the internet decreases, their violence tendency increases, which suggests that the early exposure of children, who are still developing cognitively, emotionally, and socially, to the internet increases their violence tendency.

In this study, demographic variables and variables with internet significantly affected violence tendency by 33.3% in Model 2. Variables with the internet were a significant predictor of violence tendency by an additional 27.5%. The research also found a significant relationship between internet addiction and violence tendency; as one increased, so did the other. Evli et al. found a significant relationship between internet addiction and the tendency to violence, and internet addiction predicts other variables^5^. Agbaria found a positive relationship between internet addiction and aggressive behavior in their study on high school students^10^. It can be argued that the presence of violent elements on the internet and the exposure of individuals to these elements cause them to display aggressive characteristics, become insensitive to violent behavior, and have an increased tendency toward violence.

## CONCLUSION

The violence tendency of adolescents was moderate, and internet addiction scores were low. It was determined that as the level of internet addiction and age of adolescents increased, their violence tendency increased. Internet addiction among adolescents can negatively affect their violence tendency.

This study draws attention to the relationship between violence tendency and internet addiction. Considering that age is an important factor in violence tendency, school nurses should plan and implement urgent interventions in the first years of middle school and even during primary school. They should also investigate the reasons why students have internet addiction and violence tendencies and, based on the results, take special measures to help them. Psychiatric nurses should communicate regularly with families, teachers, and students to prevent increasing internet addiction and violence. They should also plan trainings and evaluate its effectiveness to raise awareness.
